# Vitamin K2 Extends Lifespan by Alleviating Mitochondrial Stress via the JNK‐1/SIR‐2.1/DAF‐16 Signaling Axis in *Caenorhabditis elegans*

**DOI:** 10.1111/acel.70530

**Published:** 2026-05-03

**Authors:** Song‐Yu Guo, Yu‐Qi Li, Hua Piao, Wen‐Fei Zheng, Si‐Qi Li, Ze‐Yang Liu, Jun‐Ting Lv, Yue Kong, Qi‐Fa Li, Ying‐Zi Wang, Shu‐Zhuang Li, Chun‐Li Zhao, Shao Li

**Affiliations:** ^1^ Department of Physiology, College of Basic Medical Sciences, Liaoning Provincial Key Laboratory of Cerebral Diseases Dalian Medical University Dalian Liaoning China; ^2^ Department of International Medical Services Second Affiliated Hospital of Dalian Medical University Dalian China; ^3^ Department of Critical Care Medicine Affiliated Dalian Friendship Hospital of Dalian Medical University Dalian China

**Keywords:** *Caenorhabditis elegans*, extended the lifespan, JNK/SIR‐2.1/DAF‐16 pathway, mitochondrial stress, vitamin K2

## Abstract

Vitamin K2 is a fat‐soluble vitamin that has been reported to exhibit significant anti‐stress activity. Anti‐stress properties are considered to be closely associated with lifespan extension. Therefore, we investigated the effects of vitamin K2 on the lifespan and stress resistance of *Caenorhabditis elegans* , as well as the underlying mechanisms. In the present study, we found that the effects of Vitamin K2 on *C. elegans* are concentration‐dependent. High concentrations (10 μM) of Vitamin K2 are toxic to *C. elegans* , whereas lower concentrations (5 μM) are beneficial. Treatment with 5 μM Vitamin K2 can extend the lifespan of *C. elegans* , enhance its physiological functions, protect the intestinal barrier, and reduce the accumulation of lipofuscin associated with aging. Furthermore, Vitamin K2 enhanced the stress resistance of *C. elegans* by maintaining mitochondrial morphology, alleviating mitochondrial stress, reducing ROS levels, and improving mitochondrial membrane potential and ATP production. Vitamin K2 activates the JNK‐1/SIR‐2.1/DAF‐16 signaling pathway and upregulates the expression of downstream target genes such as *ctl‐1*, *ctl‐2*, *sod‐1*, *sod‐3*, and *hsp‐16.2*. We conclude that appropriate doses of Vitamin K2 protect *C. elegans* from senescence by activating the JNK‐1/SIR‐2.1/DAF‐16‐mediated anti‐mitochondrial oxidative stress pathway. These findings suggest that Vitamin K2 may have beneficial effects on lifespan and mitochondrial health in *C. elegans* , providing a basis for further investigation into its potential relevance for aging and age‐related diseases in more complex model systems.

## Graphical abstract

Vitamin K2 activates the JNK‐1/SIR‐2.1/DAF‐16 signaling axis in *C. elegans* , alleviating mitochondrial stress, reducing ROS accumulation, preserving mitochondrial function, and extending lifespan. These findings reveal a novel anti‐aging mechanism of Vitamin K2 through mitochondrial protection and stress resistance enhancement.

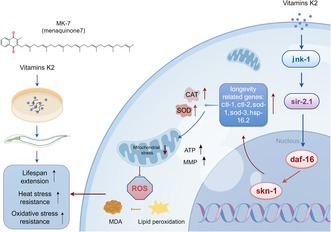

**Abbreviations**
ctl‐1: catalase‐2
ctl‐2: peroxisomal catalase 1
daf‐16: forkhead box protein O
DR: dietary restriction
FUDR: 5‐fluoro‐2′‐deoxyuridine
hsp‐16.2: heat shock protein hsp‐16.2
jnk‐1: c‐Jun N‐terminal kinase 1
MAPK: mitogen‐activated protein kinase
MDA: malondialdehyde
mtDNA: mitochondrial DNA
nsy‐1: mitogen‐activated protein kinase kinase kinase nsy‐1
pmk‐1: mitogen‐activated protein kinase pmk‐1
qRT‐PCR: quantitative real‐time PCR
ROS: reactive oxygen species
sek‐1: dual specificity mitogen‐activated protein kinase kinase sek‐1
sir‐2.1: NAD‐dependent protein deacetylase sir‐2.1
sirt‐1: sirtuin 1
skn‐1: BZIP domain‐containing protein; protein skinhead‐1
SOD: superoxide dismutase
sod‐3: superoxide dismutase [Mn] 2
UPR^MT^
: mitochondrial unfolded protein response

## 1 Introduction

Aging is an irreversible biological process in human life, characterized by a progressive decline in the structural integrity and functional capacity of physiological systems (Bettio et al. 2017; Phelps et al. 2025). This decline plays a critical role in the onset and progression of numerous age‐related diseases (Xu et al. 2015; Namba et al. 2009; Suh et al. 2007). Consequently, investigating aging mechanisms and identifying effective antiaging strategies remain a primary focus of medical inquiry and societal concern.

Mitochondria are generally regarded as the primary site for cellular energy production. Recent studies have demonstrated that mitochondria also serve as a central hub linking inflammation, oxidative stress, and aging. Reactive oxygen species (ROS) generated by the mitochondrial oxidative respiratory chain constitute the major source of intracellular ROS (Peoples et al. 2019; Son and Lee 2021). Under normal physiological conditions, appropriate levels of ROS can function as signaling molecules involved in the regulation of various cellular processes (Peoples et al. 2019). Consequently, oxidative stress‐induced imbalances in mitochondrial dynamics are considered a key initiating factor in the aging process (Miwa et al. 2022; Sharma et al. 2019). With aging, mitochondrial function progressively declines, resulting in excessive ROS production, impaired respiratory chain activity, mitochondrial DNA (mtDNA) damage, and disrupted mitophagy (Srivastava 2017; Pollard 2017; Guo, Huang, et al. 2022; Cai et al. 2022). These dysfunctional mitochondria further exacerbate oxidative stress, forming a vicious cycle that contributes to the acceleration of the aging process. Therefore, mitochondria represent the central link connecting oxidative stress and aging, and have become important targets for the treatment of age‐related diseases (Son and Lee 2021).

Vitamins are crucial nutrients indispensable for maintaining overall health and ensuring the optimal functioning of the immune system (Xie et al. 2024). Vitamin K2, which consists of a series of menaquinones (MK‐n, where *n* denotes the number of isoprenyl residues) (Schurgers and Vermeer 2000), is predominantly found in green vegetables (kale and broccoli, and certain vegetable oils like soybean oil and rapeseed oil), dairy products (Dunlop et al. 2023), and natto (Kamao et al. 2007; Bentley and Meganathan 1982; Oldenburg et al. 2008). Furthermore, it can be biosynthesized by gut microbiota; however, the quantity produced is insufficient to meet normal physiological requirements (Bentley and Meganathan 1982). Recent studies have demonstrated that Vitamin K2 may offer potential therapeutic benefits for various aging‐associated conditions, including Alzheimer's disease (Shandilya et al. 2021; Huang et al. 2021), cardiovascular disease (Khalil et al. 2021), Parkinson's disease (Prasuhn et al. 2021), among others. Although previous studies have demonstrated that vitamin K2 can extend the lifespan of *C. elegans* by enhancing fat metabolism (Qu et al. 2022), its significant protective role against mitochondrial oxidative stress during aging should not be overlooked. In this study, we aimed to further investigate the effects of vitamin K2 on lifespan and mitochondrial oxidative stress in *C. elegans* , as well as the underlying mechanisms.

## 2 Materials and Methods

### 2.1 *C. elegans* Strains and Maintenance Conditions

The following strains of *C. elegans* were acquired from the Caenorhabditis Genetics Center (University of Minnesota, MN, USA):N2, Bristol (wildtype), TJ375 (gpIs1[hsp‐16.2p::GFP]), CF1553 (muIs84[(pAD76)sod‐3p::GFP + rol‐6 (su1006)]), SJ4103 (zcIs14 [myo‐3::GFP(mit)]), TK22 (mev‐1(kn1) III), EU1(skn‐1(zu67)), KU25 (pmk‐1(km25)IV), AU3 (nsy‐1(ag3) II), AU1 (sek‐1(ag1)X), TJ356 (zIs356 [daf‐16p::daf‐16a/b::GFP + rol‐6(su1006)]), CB1370 (daf‐2(e1370) III and CF1038 daf‐16(mu86)I). VC199 (sir‐2.1(ok434) IV), VC8(jnk‐1(gk7) IV.) All *C. elegans* were cultured on NGM (Nematode Growth Medium) plates and maintained with *E. coli* OP50 as a food source.

### 2.2 Lifespan Assay

All lifespan assessments were conducted at a constant temperature of 20°C. *C. elegans* were synchronized using a hypochlorous acid solution and subsequently cultured on NGM medium. Throughout the experimental period, the animals were administered the drug daily from the egg stage until adulthood, after which they were transferred to fresh NGM medium. To prevent egg‐laying, 5‐fluoro‐2′‐deoxyuridine (FUDR) was included in the culture medium. The survival of the *C. elegans* was monitored daily, and the number of surviving individuals was recorded until all *C. elegans* in each group had expired. Mortality was confirmed by the absence of response to platinum wire stimulation. This assay was conducted independently three times, each time using at least 50 *C. elegans* per group.

### 2.3 Bending Frequency Experiment

*C. elegans* treated with 5 μM Vitamin K2 and an equivalent volume of M9 buffer were transferred to NGM plates devoid of food (*n* > 30). The bending frequency of the *C. elegans* was subsequently measured. *C. elegans* activity was quantified by counting the number of sinusoidal curves produced during a 60‐s interval in the absence of food. This assay was conducted independently three times, each time using at least 30 *C. elegans* per treatment group.

### 2.4 Beat Frequency Experiment

Several drops of M9 buffer were placed on a NGM plates, and a *C. elegans* was transferred into the droplet on the slide using a custom‐made worm pick (Guo, Guan, et al. 2022). The *C. elegans* exhibited immediate and rapid movement. The frequency of head oscillations was observed under a microscope, timed, and counted for a period of 20 s. After recording, the *C. elegans* was removed, and the process was repeated with a new nematode. 30 *C. elegans* were analyzed per experimental group. Fresh NGM plates and M9 buffer were used for each new group of experiments.

### 2.5 Pharyngeal Pump Test

A single nematode was transferred to a sterile plate free of bacteria and allowed to acclimate for approximately 2 min (Guo, Guan, et al. 2022). Subsequently, the pharynx of the nematode was observed under an inverted biological microscope equipped with a modulated objective lens, and the frequency of pharyngeal contractions was recorded over a 20‐s interval. Following the recording, the nematode was either removed from the plate or relocated to its periphery, thereby facilitating the placement of a new nematode at the center for subsequent observations. In total, 30 *C. elegans* were examined in each experimental group, and a fresh plate was utilized for each transition between groups.

### 2.6 Measurement of Body Length

Prepare a 2% agarose pad. Place the *C. elegans* intended for photography on the pad, and heat the pad over an alcohol lamp to euthanize the *C. elegans* . Subsequently, observe the *C. elegans* under an inverted microscope, measure the length of each group of *C. elegans* using a scale, and take photographs. In total, 30 *C. elegans* were examined in each experimental group, and a fresh plate was utilized for each transition between groups.

### 2.7 Body Size Assay

Following synchronization, 10 L4‐stage *C. elegans* were transferred to new NGM plates, with one nematode per plate, and were administered drugs on a daily basis. Every 12 h, they were transferred to a fresh drug‐containing culture medium until the *C. elegans* ceased egg‐laying. The number of *C. elegans* that reached adulthood was counted (excluding those that were lost, adhered, or from wormbags). The experiment was replicated three times. In total, 30 *C. elegans* were examined in each experimental group, and a fresh plate was utilized for each transition between groups.

### 2.8 Intestinal Barrier Function Assay

The integrity of the intestinal barrier was assessed using the method described in reference (Wu et al. 2023). Synchronized L4 stage *C. elegans* were cultured on NGM medium, either supplemented with or devoid of 5 μM Vitamin K2, for a period of 10 days. Subsequently, *C. elegans* were exposed to a 5% food dye FD&C Blue No. 1 (Bis[4‐(N‐ethyl‐N‐3‐sulfophenylmethyl) aminophenyl]‐2‐sulfophenylmethylium disodium salt) (Sigma‐Aldrich, USA) and incubated in M9 liquid medium for 3 h. Following this, the worms were washed with M9 buffer until the supernatant appeared clear. Intestinal barrier integrity was assessed using fluorescence inverted microscopy after anesthetizing the *C. elegans* with 20 mM levamisole. This assay was conducted independently three times, each time using at least 30 *C. elegans* per group.

### 2.9 Lipofuscin Assay

The detection of lipofuscin was conducted in accordance with the methodology described in our previous study (Wang et al. 2022). Specifically, *C. elegans* were subjected to Vitamin K2 treatment as previously detailed. On the 10th day of this regimen, the worms were transferred to 2% agarose plates and immobilized with levamisole hydrochloride (20 mM). Subsequently, the specimens were examined under an inverted fluorescence microscope (Leica DM4000B) (excitation wavelength 510–560 nm; emission wavelength 590–650 nm), and their fluorescence intensity was quantified using ImageJ. This assay was conducted independently three times, each time using at least 30 *C. elegans* per group.

### 2.10 The Stress Resistance Test

The stress resistance test followed the methodology described in previous studies (Wang et al. 2022). Synchronized L4‐stage wild‐type worms were exposed to either M9 buffer or Vitamin K2 (5 μM) for a period of 10 days prior to the stress experiments. For the heat stress experiments, the worms were transferred to fresh NGM plates and incubated at 37°C. Live and dead worms were counted until all *C. elegans* had expired. In the case of oxidative stress experiments, *C. elegans* were transferred to fresh NGM plates supplemented with 3% hydrogen peroxide (H_2_O_2_). These plates were incubated at 20°C, and live and dead worms were counted until all *C. elegans* had expired. This assay was conducted independently three times, each time using at least 30 *C. elegans* per group.

### 2.11 SOD Activity Measurement

*C. elegans* , when incubated with 5 μM Vitamin K2 and an equivalent volume of M9 buffer, were assessed for in vivo superoxide dismutase (SOD) enzyme activity utilizing a Total Superoxide Dismutase Assay Kit with WST‐8 (Beyotime, China Cat: S0101S). This assay was conducted independently three time.

### 2.12 Lipid Peroxidation MDA Assay

The Lipid Peroxidation MDA Assay Kit (Beyotime, China Cat: S0131S) was utilized to measure the oxidative status of lipids in *C. elegans* , both with and without Vitamin K2 supplementation. This assay was conducted independently three time.

### 2.13 Fat Accumulation Experiment

Fat accumulation was identified using our previously established method (Wang et al. 2022). To detect fat accumulation in senescent *C. elegans* , an Oil Red O staining kit (Solarbio, Beijing Cat:G1262) was employed. The stained samples were examined under a fluorescence microscope (Leica DM4000B), and the quantification of Oil Red O staining was performed using Image‐Pro Plus software. This assay was conducted independently three times, each time using at least 30 *C. elegans* per group.

### 2.14 Detection of ROS

The accumulation levels of ROS in *C. elegans* were quantified using a ROS Assay Kit (Beyotime, China Cat: S0033S). *C. elegans* , both treated and untreated with 5 μM Vitamin K2 for a period of 10 days, were incubated in an H_2_DCF‐DA solution prepared in M9 buffer for 30 min. Subsequently, three washes were conducted using the M9 buffer. The accumulation levels of ROS were imaged using an inverted fluorescence microscope (Leica DM4000B), and the fluorescence intensity was quantified with ImageJ software. This assay was conducted independently three times, each time using at least 30 *C. elegans* per group.

### 2.15 Determination of Mitochondrial Membrane Potential

Synchronized stage 4 wild‐type N2 worms were randomly allocated to NGM plates supplemented with Vitamin K2 or M9 buffer. The mitochondrial membrane potential was assessed using a mitochondrial membrane potential detection kit (Beyotime, China Cat: C2001S). Following a 10‐day incubation period, the worms were transferred to NGM agar plates containing TMRE (2×) and *Escherichia coli* OP50 for 12 h. Prior to imaging, the worms were incubated on standard NGM agar plates for 1 h in the dark to eliminate excess dye. Fluorescence images were captured using an inverted fluorescence microscope, and the fluorescence intensity was quantified using ImageJ software. This assay was conducted independently three times, each time using at least 30 *C. elegans* per group.

### 2.16 ATP Detection

*C. elegans* , treated with and without 5 μM Vitamin K2 for a period of 10 days, were subjected to ATP content analysis utilizing an ATP Assay Kit (Beyotime, China Cat: S0026). We conducted ATP detection in line with the instructions. To eliminate errors arising from differences in the protein amount during sample preparation, the BCA Protein Concentration Assay Kit (Beyotime, China Cat: P0009) was employed to quantify the protein in the supernatants after lysis. This assay was conducted independently three times, each time using at least 30 *C. elegans* per group.

### 2.17 Fluorescence Assay of *C. elegans*

We utilized transgenic *C. elegans* , including CF1553 (sod‐3p::GFP), TJ375 (hsp‐16.2::GFP), SJ4103 (myo‐3::GFP), LD1 (skn‐1b/c::GFP), and TJ356 (daf‐16a/b::GFP), which were subjected to either 5 μM Vitamin K2 treatment or no treatment for a period of 10 days, followed by anesthesia using 20 mM levamisole. Images were captured utilizing an inverted fluorescence microscope (Leica DM4000B), and the fluorescence intensities across different groups were quantitatively analyzed using ImageJ software.

### 2.18 *C. elegans* Mitochondrial Imaging

Mitochondrial imaging analysis was performed using the method of Petrovic et al. (2025). SJ4103 animals were maintained on either M9 or Vk2 plates until day 10. Worms were then manually selected in an M9 solution droplet, deliberately avoiding any anesthetic agents due to their strong inhibitory effects on mitochondria, and were gently immobilized using cover slips. Image acquisition was carried out with an HC PL IRAPO 40×/1.10 water‐immersion objective, utilizing excitation and emission wavelengths of 488 nm and 520 ± 20 nm, respectively. Body‐wall muscle cell images were captured from both the anterior and posterior sections of each worm, while excluding the vulval midbody region to prevent artifacts from frequent egg‐laying activity. For quantitative morphological analysis, images were processed in Fiji software with the Mosaic Suite SQUASSH plugin (for segmentation and quantification of subcellular structures), following established protocols. To enhance the computational identification of mitochondrial networks and minimize background signal, image segmentation was performed using a background removal window of 15 pixels, a regularization parameter of 0.07, and a minimum GFP intensity threshold of 0.2. Subsequently, the SQUASSH tool was employed to measure the size and perimeter of individual mitochondria. This enabled the two‐dimensional assessment of mitochondrial circularity, calculated as [circularity = (4 × π) × (Area/Perimeter^2^)], which compares object shapes to a perfect circle (where a value of 1 indicates a perfect circle and 0 a straight line). The resulting data were analyzed and visualized as Gaussian distribution plots of circularity values, using a bin size of 0.05 μm.

### 2.19 qRT‐PCR

Following a 10‐day Vitamin K2 treatment of stage 4 *C. elegans* , the samples were washed three times with M9 buffer. Total RNA was extracted using TRIzol reagent (Vazyme, China). cDNA synthesis was carried out using the HiScript II Q Select RT SuperMix for qPCR (+gDNA wiper) kit (Vazyme, China). Quantitative PCR (qPCR) was performed to analyze the expression levels of the target genes using ChamQ SYBR Color qPCR Master Mix (Vazyme, China), with β‐actin serving as the internal control.

### 2.20 Western Blot

Each group of nematodes was washed thrice with M9 solution and subsequently homogenized using a commercially available lysis buffer (Cell Signaling) supplemented with a mixture of protease and phosphatase inhibitors (Sigma). Post—homogenization, the samples underwent ultrasonic treatment and were centrifuged at 16,000 g for 5 min at 4°C. The supernatants were collected, and the protein concentration was determined using the Pierce BCA Protein Assay Kit (Thermo Scientific). Equal quantities (30 μg) of total protein from each cell extract were loaded onto SDS—PAGE gels and subjected to Western blot analysis. Proteins were separated on a 10% gradient precast polyacrylamide gel (BioRad's Mini—PROTEAN TGX StainFree Protein Gel) and then transferred to a PVDF membrane (Schleicher & Schuell, Keene). The membrane was incubated with blocking buffer (Invitrogen) for 30 min at room temperature, followed by an overnight incubation at 4°C with primary antibodies such as P—JNK (Cell Signaling Technology 9255), JNK (Sima J4500), and SIR—2.1 (Thermo Scientific PA1‐16933). After washing the membrane, signals were detected using a commercial kit (Invitrogen's Western Breeze) containing anti—rabbit or anti—mouse alkaline phosphatase—conjugated secondary antibodies and the corresponding chemiluminescent substrate, in accordance with the manufacturer's instructions. Finally, the blots were visualized using the Bio—Rad Chemidoc Touch Imaging System 732BR1030.

### 2.21 Materials

Vitamin K2 (MK‐7, water—soluble powder) was provided by Sungen Bioscience Co. Ltd. (Shantou, China). The purity of the drug was 99.63%.

### 2.22 Data and Statistical Analysis

Data are presented as mean ± standard error of the mean (SEM). For lifespan and paralysis trials, Kaplan–Meier survival curves were generated, and *p* values were calculated using the log‐rank test. Differences between two groups were assessed using the *t*‐test or χ2 test. One‐way ANOVA was used for comparisons among multiple groups. Data analysis and visualization were performed using GraphPad Prism 9.0 software (GraphPad Software Inc., La Jolla, CA, USA). *p < 0.05* was considered statistically significant.

## 3 Results

### 3.1 Vitamin K2 Extends the Lifespan of *C. elegans* and Enhances Its Physiological Functions

**FIGURE 1:**
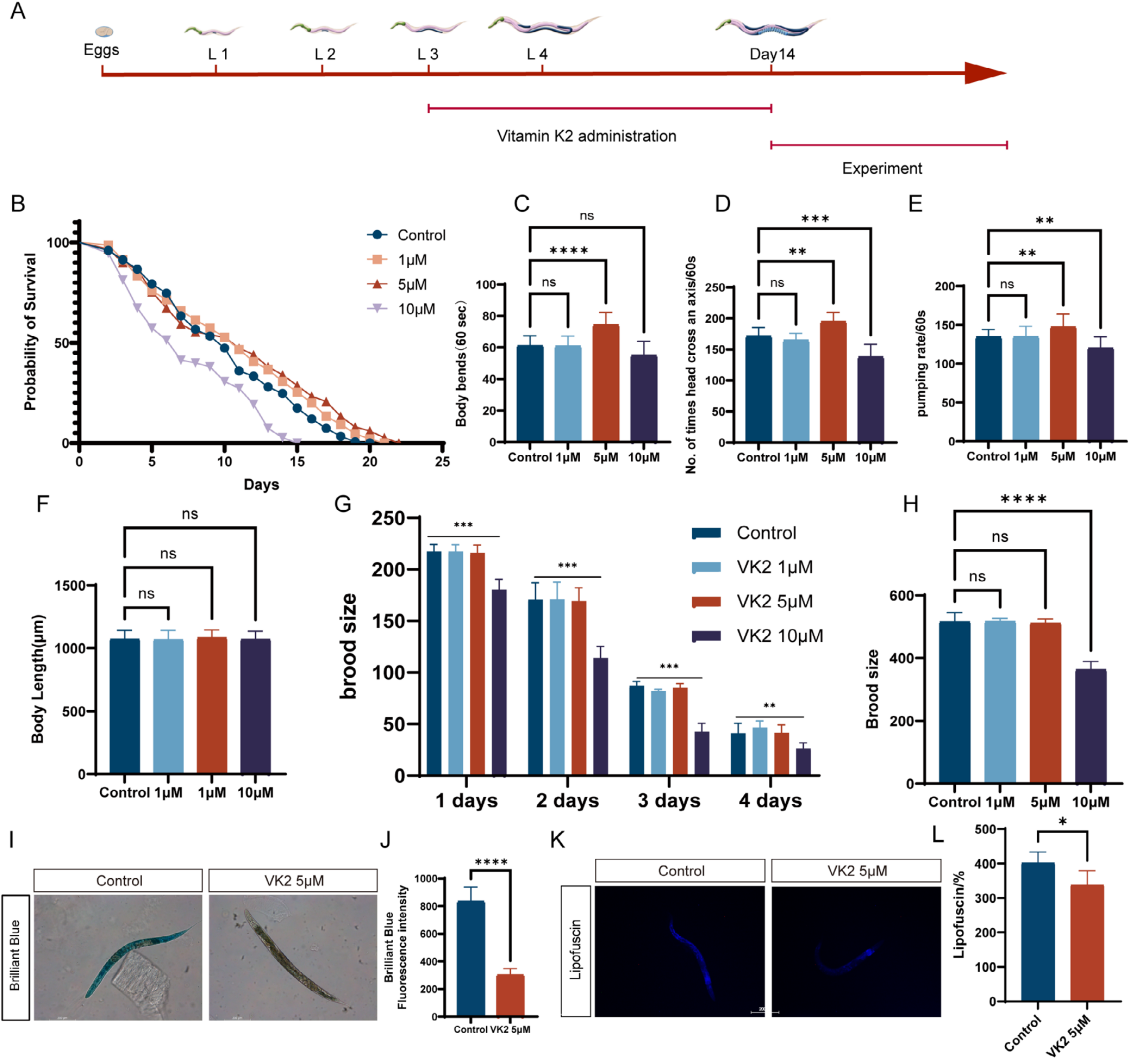
Vitamin K2 extends the lifespan of *C. elegans* and enhances its physiological functions. (A) Schematic Representation of Subsequent Experiments Following Vitamin K2 Administration. (B) The impact of a 10‐day treatment with Vitamin K2 (1, 5, 10 μM) on the lifespan of *C. elegans* . *n* = 150 in control (M9) group, *n* = 150 in Vitamin K2 (1, 5, 10 μM) treatment group (***p < 0.001, log‐rank test). (C, D) The impact of Vitamin K2 (1, 5, 10 μM) treatment on the frequency of body bending and beating in *C. elegans* at day 10. (E) The effect of Vitamin K2 (1, 5, 10 μM) treatment on pharyngeal pumping in *C. elegans* at day 10. (F) Statistical analysis of the impact of a 10‐day administration of vitamin K2 at concentrations of 1, 5, and 10 μM on the body length of *C. elegans* . (G, H) Statistical analysis of the effects of vitamin K2 (1, 5, and 10 μM) on the fecundity of *Caenorhabditis elegans* after a 10‐day treatment. (G) The number of eggs laid from the first to the fourth day of adulthood. (H) The total number of eggs laid by nematodes. (I, J) The Impact of Vitamin K2 (5 μM) Treatment on the Intestinal Barrier of *C. elegans* at Day 10. (K, L) Impact of Vitamin K2 (5 μM) Administration on Intestinal Lipofuscin Levels in *C. elegans* on Day 10. The summarized data are presented in the right panel. Total fluorescence per worm was analyzed using ImageJ software. The data of the two groups were analyzed by *T*‐test. One‐way ANOVA was used for comparisons among multiple groups. Values are presented as mean ± SEM. All of these measurements were made at least three times. *p < 0.05, **p < 0.01, ***p < 0.001.

To evaluate the impact of Vitamin K2 on the lifespan of *C. elegans* , we initially optimized the dosing protocol of Vitamin K2 in *C. elegans* . The *C. elegans* were exposed to various concentrations of Vitamin K2 for a period of 10 days, after which their lifespans were assessed (Figure 1A,B). Treatment with 10 μM Vitamin K2 resulted in a significant reduction in the lifespan of *C. elegans* when compared to the control group (Figure 1B; median survival: Control:10, 10 μM: 7; Hazard Ratio:1.724; 95% CI of ratio:1.363 to 2.180). Conversely, treatments with 1 μM and 5 μM Vitamin K2 led to a notable extension of the lifespan of *C. elegans* relative to the control (Figure 1B; median survival: Control:10, 1 μM: 11, 5 μM: 11; Hazard Ratio: 1 μM: 0.8106, 5 μM: 0.7534; 95% CI of ratio: 1 μM: 0.6456 to 1.018, 5 μM: 0.5995 to 0.9469). Furthermore, exposure to 5 μM Vitamin K2 enhanced the survival rate of *C. elegans* in later stages of life more effectively than a 10‐day treatment with 1 μM Vitamin K2 (Figure 1B).

To further determine the optimal dosage concentration of vitamin K2 for anti—aging purposes and whether it can enhance the health status of the subjects, we subsequently carried out additional measurements of their physiological vitality. These measurements included indicators such as the movement ability, pharyngeal pumping ability, body length, and reproductive capacity of the *C. elegans* .

During the aging process, *C. elegans* exhibits a progressive decline in motility (Zheng et al. 2024). To quantify this change, we utilized the frequency of body bending (number of body bends per 60s on NGM and in M9 solution) as an index of motility. Our results demonstrated that a 10‐day treatment with 5 μM Vitamin K2 significantly mitigated the decline in motility compared to the control group. In contrast to the control group, a concentration of 1 μM vitamin K2 did not enhance the locomotion ability of *C. elegans* , whereas a concentration of 10 μM vitamin K2 diminished their locomotion ability (Figure 1C,D).

The frequency of the pharyngeal pump is intricately linked to the aging process. With advancing age, the frequency of the pharyngeal pump progressively diminishes. Our findings demonstrated a statistically significant enhancement in the pharyngeal pump rate in the 5 μM Vitamin K2 group relative to the control group. However, a concentration of 1 μM vitamin K2 did not enhance the pharyngeal pump frequency, whereas a concentration of 10 μM vitamin K2 decreased this phenomenon (Figure 1E).

Subsequently, we also investigated the changes in the body length of *C. elegans* . Our findings demonstrated that after feeding the worms with 1 μM, 5 μM, and 10 μM of vitamin K2 for a period of 10 days, there was no change in their body length when compared to the control group (Figure 1F).

The results of the fertility determination experiment indicated that, in comparison with the control group, 1 μM and 5 μM of vitamin K2 had no impact on the number of eggs laid by the *C. elegans* , whereas 10 μM of vitamin K2 resulted in a reduction in the number of eggs laid by *C. elegans* (Figure 1G,H).

In conclusion, our findings indicate that 5 μM vitamin K2 is more effective than 1 μM vitamin K2 in enhancing normal physiological functions. Additionally, it appears that 10 μM vitamin K2 is detrimental to the growth and development of *C. elegans* . To validate this, we conducted further investigations into the accumulation of reactive oxygen species (ROS), adenosine triphosphate (ATP) content, and mitochondrial morphology in the *C. elegans* . Our results demonstrated that 5 μM vitamin K2 significantly decreased the level of ROS accumulation in *C. elegans* and notably increased the ATP content. Conversely, 10 μM vitamin K2 resulted in a higher level of ROS accumulation compared to the control group and also led to a reduction in the ATP content in *C. elegans* (Figure S1A–C). We compared the mitochondrial networks in SJ4103[Pmyo—3::mitoGFP] animals with GFP expressed in the body—wall mitochondria. It was observed that, in aged animals, a 5 μM treatment led to fewer mitochondrial fragments and longer mitochondria when compared to the untreated group. Conversely, a 10 μM treatment in aged animals resulted in more mitochondrial fragments and shorter mitochondria as compared to the untreated group (Figure S1D,E). Therefore, we selected 5 μM of vitamin K2 for the subsequent experimental investigation.

The maintenance of intestinal barrier integrity is crucial for the health of *C. elegans* , and intestinal permeability has been observed to increase with age (Dambroise et al. 2016; McGee et al. 2011). The intestinal integrity of *C. elegans* was assessed using the food dye FD&C Blue No. 1. As shown, a 10‐day treatment with Vitamin K2 significantly reduced coelom leakage in worms compared to the control group (Figure 1I,J). Lipofuscin, an auto fluorescent “age pigment” commonly utilized as an indicator of aging in worms, accumulates progressively with age (Zheng et al. 2024). A 10‐day administration of Vitamin K2 inhibited the accumulation of lipofuscin in the intestines of *C. elegans* relative to the control group (Figure 1K,L).

### 3.2 Vitamin K2 Augments the Stress Resistance of *C. elegans* Through the Enhancement of Mitochondrial Function

**FIGURE 2:**
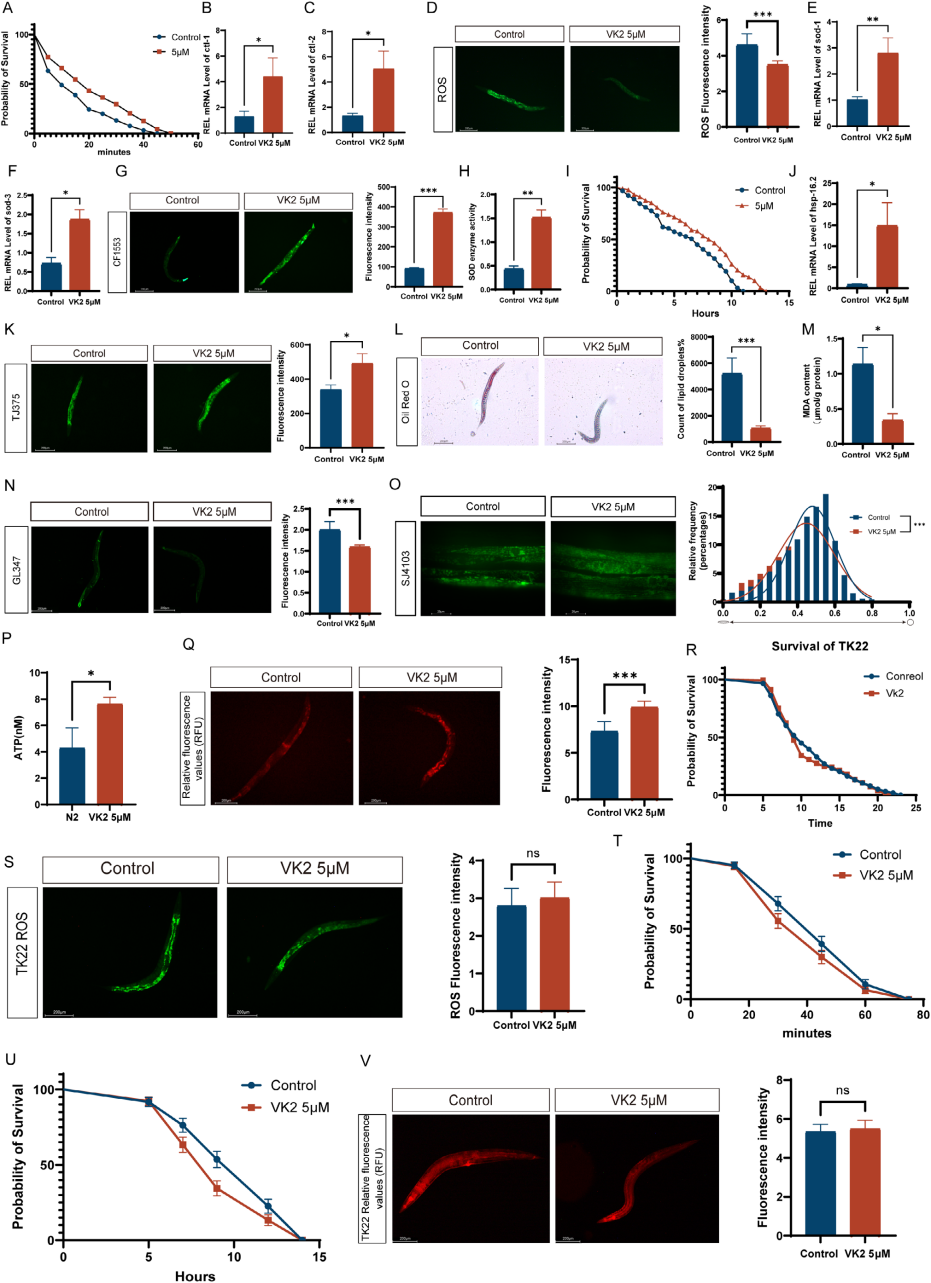
Vitamin K2 augments the stress resistance of *C. elegans* through the enhancement of mitochondrial function. (A) Vitamin K2 (5 μM) was administered to *C. elegans* under conditions of oxidative stress induced by 3% H_2_O_2_. *n* = 90 in control (M9) group, *n* = 90 in Vitamin K2 (5 μM) treatment group (***p < 0.001, log‐rank test). (B, C) *ctl‐1*, *ctl‐2* mRNA levels in worms treated with 5 μM Vitamin K2 and controls. (D) Effects of 5 μM Vitamin K2 on ROS levels in wild‐type worms. (E, F) *sod‐1*, *sod‐3* mRNA levels in worms treated with 5 μM Vitamin K2 and controls. (G) Fluorescence images of CF1553 *C. elegans* treated with 5 μM Vitamin K2, and untreated. (H) The total SOD enzyme activity was measured in *C. elegans* exposed to 5 μM Vitamin K2 and in the untreated control group. (I) The mean survival time of wild‐type worms treated with 5 μM Vitamin K2 at 37°C. *n* = 90 in control (M9) group, *n* = 90 in Vitamin K2 (5 μM) treatment group (***p < 0.001, log‐rank test). (J) *hsp‐16.2* mRNA levels in worms treated with 5 μM Vitamin K2 and controls. (K) Fluorescence images of TJ375 *C. elegans* treated with 5 μM Vitamin K2, and untreated. (L) The effect of Vitamin K2 on fat accumulation. (M) MDA content in *C. elegans* in 5 μM Vitamin K2 and untreated groups. (N) Expression and Quantification of UPR^MT^ (hsp‐6p::GFP) in *C. elegans* (O) The effect of 5 μM vitamin K2 treatment on mitochondrial morphology in the body wall muscle of *C. elegans* and an analysis of mitochondrial roundness. (P) Vitamin K2 (5 μM) treatment for 10 days increased ATP levels. (Q) Vitamin K2 (5 μM) treatment for 10 days increased mitochondrial membrane potential (MMP) in worms. (R) Effects of 5 μM Vitamin K2 untreated treatments on the lifespan of TK22. (***p < 0.001, log‐rank test) (S) Effects of 5 μM Vitamin K2 on ROS levels in TK22. (T) Vitamin K2 (5 μM) was administered to TK22 under conditions of oxidative stress induced by 3% H_2_O_2_. *n* = 90 (U) The mean survival time of TK22 treated with 5 μM Vitamin K2 at 37°C. *n* = 90 (V) Vitamin K2 (5 μM) treatment for 10 days reduced mitochondrial membrane potential (MMP) in TK22. Total fluorescence per worm was analyzed using ImageJ software. Data were analyzed by T‐test using Prism 9.0. Values are presented as mean ± SEM; All of these measurements were made at least three times. *p < 0.05, **p < 0.01, ***p < 0.001.

Research has demonstrated a significant correlation between the stress resistance of *C. elegans* and its lifespan (Shen et al. 2021). To investigate whether Vitamin K2 can enhance stress resistance in *C. elegans* , we conducted an acute oxidative stress assay. The results demonstrated that Vitamin K2 (5 μM) significantly improved the resistance of *C. elegans* to H_2_O_2_‐induced oxidative stress (Figure 2A Median survival: Control:10, 5 μM: 20; Hazard Ratio:0.6719; 95% CI of ratio:0.4982 to 0.9060). *ctl‐1* and *ctl‐2* are responsible for initiating catalase activity in *C. elegans* . Our findings demonstrate that Vitamin K2 significantly enhances the expression levels of *ctl‐1* and *ctl‐2* in *C. elegans* (Figure 2B,C).

The routine metabolic processes of cells lead to a significant accumulation of ROS, and elevated levels of ROS have been documented to contribute to the aging process (Li et al. 2003; Gusarov et al. 2021). Consequently, we investigated the impact of a 5 μM Vitamin K2 treatment on ROS levels in senescent N2 wild‐type *C. elegans* . Our findings demonstrated that the administration of Vitamin K2 for 10 days resulted in a reduction of ROS accumulation (Figure 2D). Superoxide dismutase (SOD) exhibits antioxidant and anti‐aging properties, primarily through the scavenging of harmful oxygen free radicals (Shao et al. 2023). To investigate whether the reduction of ROS levels in *C. elegans* by Vitamin K2 is associated with SOD, the mRNA expression levels of *sod‐1* and *sod‐3* were assessed. Our findings indicated that Vitamin K2 significantly upregulated the expression of both *sod‐1* and *sod‐3* in *C. elegans* (Figure 2E,F). To validate these results, we employed the transgenic strain *C. elegans* CF1553 to monitor the expression of the *sod‐3* gene. The data confirmed that Vitamin K2 augmented *sod‐3* expression in aging *C. elegans* (Figure 2G). Furthermore, the total SOD activity in *C. elegans* was quantified. The findings indicated that Vitamin K2 administration significantly enhanced the overall SOD enzymatic activity in the *C. elegans* (Figure 2H). Heat tolerance can serve as an indicator of aging in *C. elegans* (Gusarov et al. 2021). We investigated whether 5 μM Vitamin K2 could enhance heat tolerance in aging *C. elegans* . Our results demonstrate that under heat stress conditions, 5 μM Vitamin K2 significantly shifted the survival curve to the right and improved heat tolerance in *C. elegans* compared to the control group (Figure 2I Median survival: Control: 7, 5 μM:8.250; Hazard Ratio:0.6209; 95% CI of ratio: 0.4587 to 0.8403). Subsequently, we quantified the mRNA expression levels of the heat shock protein *hsp‐16.2* using quantitative real‐time PCR (qRT‐PCR) and assessed the expression of hsp‐16.2 in transgenic *C. elegans* strain TJ375. The findings indicated that Vitamin K2 markedly enhanced the expression of the heat shock protein *hsp‐16.2* (Figure 2J,K).

In *C. elegans* , the excessive accumulation of ROS can result in an increase in fat deposition. Furthermore, accumulation is intricately linked to the aging process (Yu et al. 2021). We investigated fat accumulation in *C. elegans* through Oil Red O staining. The results demonstrated that Vitamin K2 can significantly decrease the lipid accumulation levels in *C. elegans* (Figure 2L). In vivo, free radicals induce lipid peroxidation, with the end product being malondialdehyde (MDA) (Hui et al. 2020). MDA can facilitate the cross‐linking and polymerization of proteins, nucleic acids, and other macromolecules, exhibiting cytotoxic effects (Hui et al. 2020). We conducted an examination of MDA levels in *C. elegans* . The results indicated that Vitamin K2 significantly reduced MDA levels in the nematode population (Figure 2M). Aging can induce activation of the mitochondrial unfolded protein response (UPR^MT^). However, prolonged activation of UPR^MT^ is detrimental to cellular function (Martinez et al. 2017). We utilized the GL347 strain of *C. elegans* to investigate the in vivo activation of the mitochondrial unfolded protein response. Our findings demonstrated that UPR^MT^ was indeed activated in aged (senescent) *C. elegans* , and that supplementation with vitamin K2 significantly attenuated UPR^MT^ activation in *C. elegans* (Figure 2N). Structural degeneration of mitochondria serves as a key marker of cellular senescence in *C. elegans* (Yavorov‐Dayliev et al. 2023). We conducted a comparison of the mitochondrial networks in SJ4103[Pmyo—3::mitoGFP] animals, which express GFP in the body—wall mitochondria. It was observed that, when compared to untreated aged animals, those treated with 5 μM vitamin K2 exhibited fewer mitochondrial fragments and longer mitochondria (Figure 2O, Figure S1D,E). This finding accounts for the significant impact of vitamin K2 on motility. The ATP content test indicated that vitamin K2 was capable of increasing the ATP level of senescent *C. elegans* (Figure 2P). Moreover, the results of TMRE staining demonstrated that the treatment with vitamin K2 enhanced the mitochondrial membrane potential in senescent nematodes (Figure 2Q). To further explore the relationship between vitamin K2‐induced mitochondrial stress and aging, we utilized the mitochondrial respiratory chain mutant nematode strain TK22. Our results indicated that the mev—1 mutation nullified the beneficial impact of vitamin K2 on *C. elegans* . Following the mev—1 mutation, vitamin K2 was no longer able to extend the lifespan of *C. elegans* (Figure 2R Median survival: Control:10, 5 μM:9; Hazard Ratio:1.053; 95% CI of ratio:0.8394 to 1.320), and it could not reduce the accumulation of ROS (Figure 2S). Furthermore, the mev—1 mutant was unable to enhance resistance to acute oxidative stress (Figure 2T Median survival: Control:45, 5 μM:45; Hazard Ratio:1.182; 95% CI of ratio:0.8775 to 1.591) and heat stress (Figure 2U Median survival: Control:12, 5 μM:9; Hazard Ratio:1.288; 95% CI of ratio:0.9554 to 1.735), nor could it regulate the mitochondrial membrane potential in aged *C. elegans* (Figure 2V). These findings imply that a functional mev—1 gene is necessary for vitamin K2 to exert its effect on extending the nematode lifespan.

### 3.3 Vitamin K2 Promotes Stress Resistance/Longevity via Skn‐1

**FIGURE 3:**
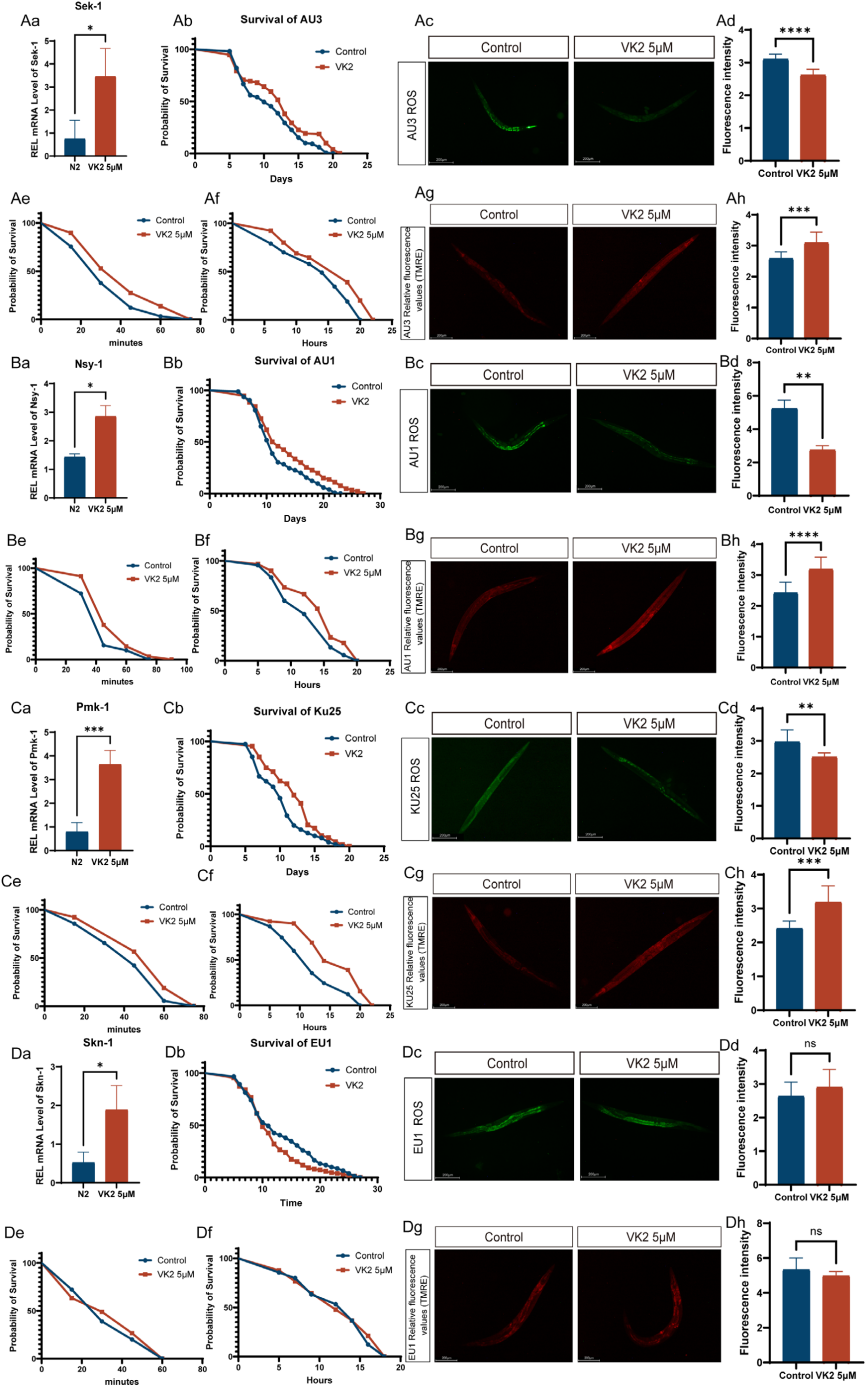
Vitamin K2 promotes stress resistance/longevity via skn‐1. (Aa) *sek‐1* mRNA levels in worms treated with 5 μM Vitamin K2 and controls. (Ab) Effects of 5 μM Vitamin K2 treated treatments on the lifespan of AU3 *C. elegans* . (*p < 0.05, log‐rank test) (Ac, Ad) Effects of 5 μM Vitamin K2 on ROS levels in AU3 *C. elegans* . (Ae) Vitamin K2 (5 μM) was administered to AU3 under conditions of oxidative stress induced by 3% H_2_O_2_. (Af) The mean survival time of AU3 treated with 5 μM Vitamin K2 at 37°C. (Ag, Ah) Vitamin K2 (5 μM) treatment for 10 days reduced mitochondrial membrane potential (MMP) in AU3. (Ba) *nsy‐1* mRNA levels in worms treated with 5 μM Vitamin K2 and controls. (Bb) Effects of 5 μM Vitamin K2 treated treatments on the lifespan of AU1 *C. elegans* . (Bc, Bd) Effects of 5 μM Vitamin K2 on ROS levels in AU1 *C. elegans* . (Be) Vitamin K2 (5 μM) was administered to AU1 under conditions of oxidative stress induced by 3% H_2_O_2_. (Af) The mean survival time of AU1 treated with 5 μM Vitamin K2 at 37°C. (Bg, Bh) Vitamin K2 (5 μM) treatment for 10 days reduced mitochondrial membrane potential (MMP) in AU1. (Ca) *pmk‐1* mRNA levels in worms treated with 5 μM Vitamin K2 and controls. (Cb) Effects of 5 μM Vitamin K2 treatments on the lifespan of KU25 *C. elegans* (*p < 0.05, log‐rank test). (Cc, Cd) Effects of 5 μM Vitamin K2 on ROS levels in Ku25 *C. elegans* . (Ce) Vitamin K2 (5 μM) was administered to Ku25 under conditions of oxidative stress induced by 3% H_2_O_2_. (Cf) The mean survival time of Ku25 treated with 5 μM Vitamin K2 at 37°C. (Cg, Ch) Vitamin K2 (5 μM) treatment for 10 days reduced mitochondrial membrane potential (MMP) in Ku25. (Da) *skn‐1* mRNA levels in worms treated with 5 μM Vitamin K2 and controls. (Db) Effects of 5 μM Vitamin K2 untreated treatments on the lifespan of EU1 *C. elegans* (*p < 0.005, log‐rank test). (Dc, Dd) Effects of 5 μM Vitamin K2 on ROS levels in EU1 *C. elegans* . (De) Vitamin K2 (5 μM) was administered to EU1 under conditions of oxidative stress induced by 3% H_2_O_2_. (Df) The mean survival time of Ku25 treated with 5 μM Vitamin K2 at 37°C. (Dg, Dh) Vitamin K2 (5 μM) treatment for 10 days reduced mitochondrial membrane potential (MMP) in EU1. Total fluorescence per worm was analyzed using ImageJ software. Data were analyzed by *T*‐test using Prism 9.0. Values are presented as mean ± SEM; All of these measurements were made at least three times. *p < 0.05, ***p < 0.001.

The transcription factor skn‐1 is essential for the induction of genes involved in ROS detoxification and the promotion of longevity (Liu, Wang, Zhu, et al. 2022). ROS can function as signaling molecules that activate skn‐1 via the p38/PMK‐1 signaling pathway (Soltanmohammadi et al. 2022). The expression levels of *sek‐1*, *nsy‐1*, and *pmk‐1*, which are key molecules in the PMK‐1/p38 signal pathway, were investigated in *C. elegans* following a 10‐day treatment with 5 μM Vitamin K2. The mRNA expression levels of *sek‐1*, *nsy‐1*, and *pmk‐1* were significantly elevated as a result of the Vitamin K2 treatment (Figure 3Aa,Ba,Ca). We further elucidated the role of PMK‐1/p38 MAPK signal pathway in Vitamin‐induced lifespan extension in *C. elegans* by utilizing *sek‐1*, *nsy‐1*, and *pmk‐1* mutant strains. Our findings demonstrated that Vitamin K2 continued to extend the lifespan of *sek‐1* (Figure 3Ab Median survival: Control:10, 5 μM:13; Hazard Ratio:0.7308; 95% CI of ratio:0.55812 to 0.9190), *nsy‐1* (Figure 3Bb Median survival: Control:11, 5 μM:11.5; Hazard Ratio:0.7037; 95% CI of ratio:0.5600 to 0.8842), and *pmk‐1* mutants (Figure 3Cb Median survival: Control:10, 5 μM:12; Hazard Ratio:0.6510; 95% CI of ratio:0.5171 to 0.8196). The effects of vitamin K2 on the stress resistance of *C. elegans* mutants were investigated. The results showed that vitamin K2 could still reduce the ROS levels in sek‐1, nsy‐1, and pmk‐1 mutant *C. elegans* (Figure 3Ac,Ad,Bc,Bd,Cc,Cd). It could also enhance the resistance of mutant *C. elegans* to acute oxidative stress and acute heat stress (Figure 3Ae Median survival: Control:30, 5 μM:45; Hazard Ratio: 0.7094; 95% CI of ratio:0.5265 to 0.9558, Af Median survival: Control: 14, 5 μM:18; Hazard Ratio: 0.5983; 95% CI of ratio:0.4424 to 0.8091, Be Median survival: Control:45, 5 μM:45; Hazard Ratio:0.4496; 95% CI of ratio:0.5390 to 0.9743, Bf Median survival: Control:12, 5 μM:16; Hazard Ratio:0.7734; 95% CI of ratio:0.5761 to 1.038, Ce Median survival: Control:45, 5 μM:60; Hazard Ratio: 0.7028; 95% CI of ratio:0.5223 to 0.9456, Cf Median survival: Control:12, 5 μM:14; Hazard Ratio:0.5226; 95% CI of ratio:0.3842 to 0.7109), as well as increase the mitochondrial membrane potential levels of mutant *C. elegans* (Figure 3Ag,Ah,Bg,Bh,Cg,Ch). These results suggest that although vitamin K2 upregulated the expression levels of sek‐1, nsy‐1, and pmk‐1 in aging *C. elegans* , it is not indispensable for the enhancement of stress resistance or the extension of lifespan in *C. elegans* .

The transcription factor skn‐1 is capable of regulating mitochondrial function, a process that plays a critical role in neuroprotection and longevity (Lautrup et al. 2019; Blackwell et al. 2015; Tullet et al. 2017). Our findings demonstrate that vitamin K2 enhances the expression level of skn‐1 in aging *C. elegans* (Figure 3Da). Subsequently, we employed the skn‐1 mutant *C. elegans* strain EU1 to further investigate the involvement of skn‐1 in mediating the effects of vitamin K2 on stress resistance and lifespan extension in *C. elegans* . Our results demonstrated that vitamins were ineffective in extending the lifespan of skn‐1 mutant *C. elegans* (Figure 3Db Median survival: Control:11, 5 μM:10; Hazard Ratio:1.260; 95% CI of ratio:1.004 to 1.583), nor did they reduce intracellular ROS levels (Figure 3Dc,Dd). Furthermore, vitamins failed to enhance resistance to acute oxidative and heat stress (Figure 3De Median survival: Control:30, 5 μM:30; Hazard Ratio:0.9211; 95% CI of ratio:0.6875 to 1.234, Df Median survival: Control: 14, 5 μM:12; Hazard Ratio:0.9306; 95% CI of ratio:0.6947 to 1.247), and showed no significant effect on mitochondrial membrane potential in the mutant *C. elegans* (Figure 3Dg,Dh). In conclusion, skn‐1 may serve as a key mediator through which vitamin K2 regulates stress response and extends lifespan in *C. elegans* .

### 3.4 Vitamin K2 Extended the Lifespan of *C. elegans* by Mitigating Mitochondrial Stress via Activation of the JNK/SIR‐2.1/DAF‐16 Pathway

**FIGURE 4:**
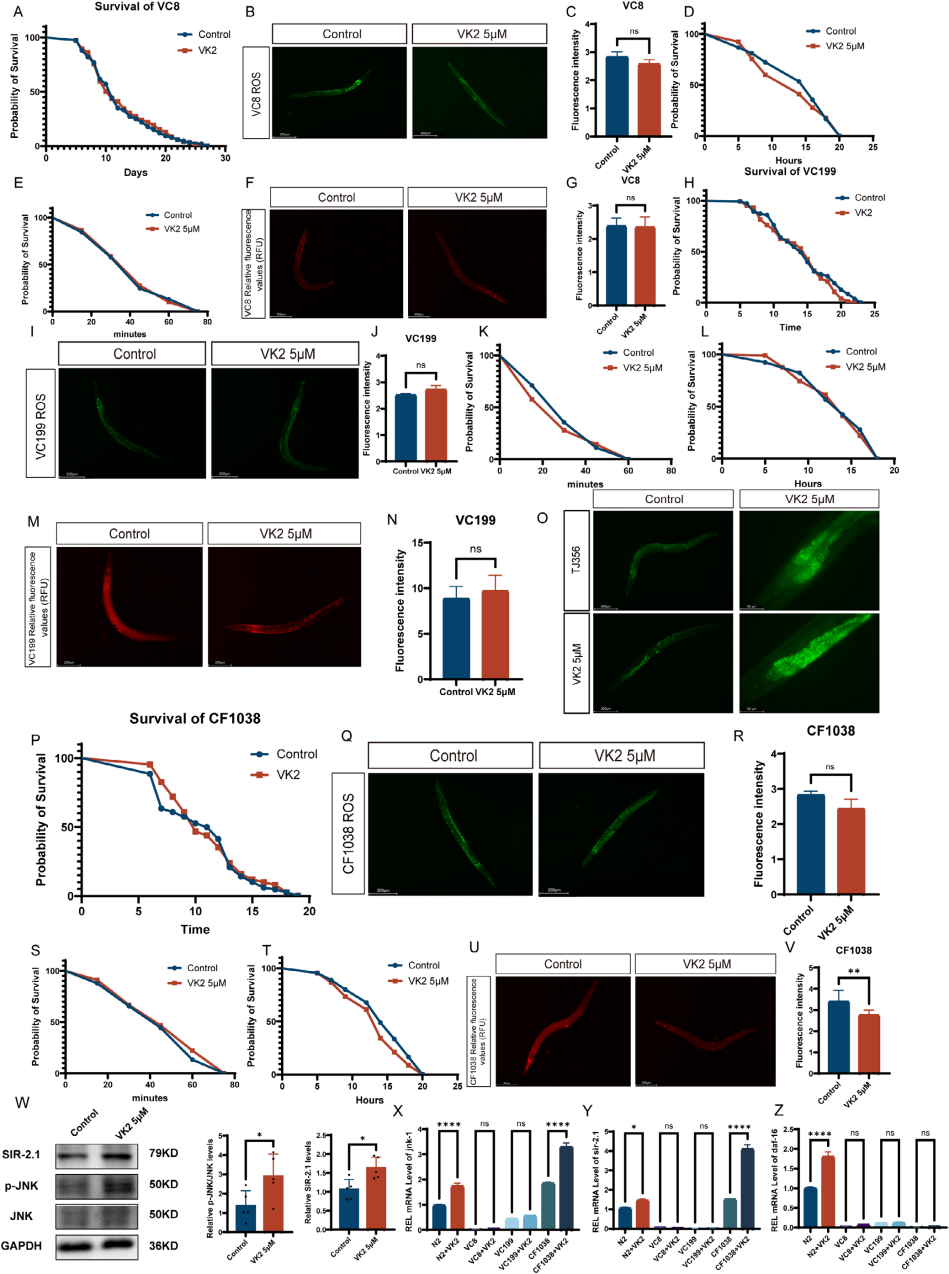
Vitamin K2 extended the lifespan of *C. elegans* by mitigating mitochondrial stress via activation of the JNK/SIR‐2.1/DAF‐16 Pathway. (A) Effects of 5 μM Vitamin K2 untreated treatments on the lifespan of VC8 *C. elegans* (log‐rank test). (B, C) Effects of 5 μM Vitamin K2 on ROS levels in VC8 *C. elegans* . (D) Vitamin K2 (5 μM) was administered to VC8 under conditions of oxidative stress induced by 3% H_2_O_2_. (E) The mean survival time of VC8 treated with 5 μM Vitamin K2 at 37°C. (F, G) Vitamin K2 (5 μM) treatment for 10 days reduced mitochondrial membrane potential (MMP) in VC8. (H) Effects of 5 μM Vitamin K2 untreated treatments on the lifespan of VC199 *C. elegans* (log‐rank test). (I, J) Effects of 5 μM Vitamin K2 on ROS levels in VC199 *C. elegans* . (K) Vitamin K2 (5 μM) was administered to VC199 under conditions of oxidative stress induced by 3% H_2_O_2_. (L) The mean survival time of VC199 treated with 5 μM Vitamin K2 at 37°C. (M, N) Vitamin K2 (5 μM) treatment for 10 days reduced mitochondrial membrane potential (MMP) in VC199. (O) Treatment with Vitamin K2 (5 μM) for 10 days resulted in an increase in Daf‐16: GFP nuclear translocation in *C. elegans* . (P) Effects of 5 μM Vitamin K2 untreated treatments on the lifespan of CF1038 *C. elegans* (log‐rank test). (Q, R) Effects of 5 μM Vitamin K2 on ROS levels in CF1038 *C. elegans* . (S) Vitamin K2 (5 μM) was administered to CF1038 under conditions of oxidative stress induced by 3% H_2_O_2_. (T) The mean survival time of CF1038 treated with 5 μM Vitamin K2 at 37°C. (U, V) Vitamin K2 (5 μM) treatment for 10 days reduced mitochondrial membrane potential (MMP) in CF1038. (W) Western blot images and statistical graphs of p‐JNK, JNK, and SIR‐2.1 proteins following a 10‐day treatment with vitamin K2 (5 μmol). (X, Y, Z) The mRNA expression levels of *jnk—1*, *sir—2.1*, and *daf—16* in N2, VC8, VC199, and CF1038 strains of *C. elegans* were measured after a 10‐day treatment with vitamin K2 at a concentration of 5 μmol. The data of the two groups were analyzed by *T*‐test. One‐way ANOVA was used for comparisons among multiple groups. Values are presented as mean ± SEM. All of these measurements were made at least three times. *p < 0.05, **p < 0.01, ***p < 0.001.

In *C. elegans* , numerous studies have underscored the role of the JNK signal pathway in a wide array of cellular functions, including developmental regulation (Smith et al. 2002), neuronal regeneration (Li et al. 2012), and stress response modulation (Gerke et al. 2014). The *jnk‐1* mutant nematode strain VC8 was utilized to investigate the role of Vitamin K2 in extending the lifespan of *C. elegans* . The results indicated that administration of 5 μM Vitamin K2 did not result in an extended lifespan for the VC8 *C. elegans* (Figure 4A; median survival: Control: 11, 5 μM:11; Hazard Ratio: 0.9760; 95% CI of ratio:0.7819 to 1.228). Vitamin K2 did not reduce ROS levels in the jnk‐1 mutant *C. elegans* (Figure 4B,C), failed to enhance the mutant nematode's resistance to acute oxidative and heat stress (Figure 4D; median survival: Control: 16, 5 μM:14; Hazard Ratio: 1.107; 95% CI of ratio:0.8260 to 1.483, E Median survival: Control:45, 5 μM:45; Hazard Ratio:1.002; 95% CI of ratio:0.7479 to 1.342), and did not improve mitochondrial membrane potential in the organism (Figure 4F,G). These findings suggest that the activation of *jnk‐1* is necessary for Vitamin K2 to exert its lifespan‐prolonging effects on *C. elegans* . *sirt‐1* functions as a cellular survival factor, enhancing the resistance of mammalian cells to oxidative stress (Nasrin et al. 2009). Jnk‐1 phosphorylates sirt‐1, thereby promoting its enzymatic activity (Nasrin et al. 2009). We conducted a more in‐depth investigation into the role of *sir‐2.1* in the Vitamin K2‐induced lifespan extension in *C. elegans* by utilizing *sir‐2.1* mutant worms. The results demonstrated that treatment with Vitamin K2 (5 μM) did not extend the lifespan of *sir‐2.1* mutant worms when compared to the control group (Figure 4H; median survival: Control:14, 5 μM:15; Hazard Ratio:1.195; 95% CI of ratio:0.9524 to 1.500). Vitamin K2 was unable to decrease ROS levels in sir‐2.1 mutant *C. elegans* (Figure 4I,J), showed no effect on enhancing the mutant's tolerance to acute oxidative and thermal stress (Figure 4K Median survival: Control: 30, 5 μM:30; Hazard Ratio:1.084; 95% CI of ratio:0.8094 to 1.453, L Median survival: Control:14, 5 μM:14; Hazard Ratio: 1.071; 95% CI of ratio: 0.7992 to 1.434), and did not lead to an improvement in mitochondrial membrane potential in sir‐2.1 mutant *C. elegans* (Figure 4M,N). Daf‐16, a direct homolog of the mammalian FOXO transcription factor, is a key pro‐longevity transcription factor integrating multiple longevity pathways (Sun et al. 2017). *Sir‐2.1* binds to *daf‐16* in the Insulin/IGF‐1 signal pathway (IIS) and promotes its activation (Sun et al. 2017) We utilized TJ356 (daf‐16::GFP) worms to evaluate the nuclear translocation of *Daf‐16* following Vitamin K2 treatment. The results demonstrated that a 10‐day treatment with Vitamin K2 (5 μM) significantly enhanced the nuclear translocation of *daf‐16* in *C. elegans* compared to the control group (Figure 4O). In *Daf‐16* mutant *C. elegans* , Vitamin K2 treatment failed to extend lifespan compared with controls (Figure 4P Median survival: Control: 11.5, 5 μM:10; Hazard Ratio: 0.9445; 95% CI of ratio:0.7525 to 1.185). Vitamin K2 did not reduce reactive oxygen species levels in *Daf‐16* mutant *C. elegans* (Figure 4Q,R), nor did it enhance resistance to acute oxidative or heat stress in these mutant *C. elegans* (Figure 4S Median survival: Control:45, 5 μM:45; Hazard Ratio:0.8916; 95% CI of ratio:0.6654 to 1.195, T Median survival: Control: 14, 5 μM:14; Hazard Ratio:1.260; 95% CI of ratio:0.9386 to 1.690). Additionally, no improvement was observed in mitochondrial membrane potential (Figure 4U,V). In jnk—1, sir—2.1, and daf—16 mutants, vitamin K2 did not extend their lifespan. To rule out the possibility that the ineffectiveness was due to defects in digestion and absorption in these mutants, we further examined several basic physiological functions of these mutants after the first day of adulthood, including locomotor ability, pharyngeal pumping rate, egg—laying rate, and body length. The results indicated that these mutants exhibited no obvious developmental defects (Figure S2). To further validate that vitamin K2 can activate the expression of JNK, a Western blot analysis was conducted to measure the phosphorylation level of JNK in wild—type N2 worms that were treated with 5 μM vitamin K2 for 10 days. As depicted in the figure, the treatment with vitamin K2 significantly elevated the ratio of phosphorylated JNK (p—JNK) to total JNK when compared with the control worms (treated with M9) (*p* < 0.05) (Figure 4W). This suggests that vitamin K2 promotes the activation of JNK via phosphorylation. Subsequently, the protein expression of SIR—2.1 was also examined, and the results, as shown in the figure, demonstrated that vitamin K2 significantly upregulated the expression of SIR—2.1 (Figure 4W). To further investigate whether vitamin K2 mediates its effects via the jnk‐1/sir −2.1/daf‐16 signaling cascade, we measured the expression levels of jnk‐1, sir‐2.1, and daf‐16 in N2, VC8, VC199, and CF1038 strains of *C. elegans* following vitamin K2 administration. The results demonstrated that following vitamin K2 administration, the expression levels of jnk‐1, sir‐2.1, and daf‐16 genes were upregulated in N2 wild‐type *C. elegans* (Figure 4X–Z). In the jnk‐1 mutant, the expression levels of sir‐2.1 and daf‐16 were downregulated. In sir‐2.1 mutants, jnk‐1 expression exhibited a slight increase, whereas daf‐16 expression was reduced. In the daf‐16 mutant, the expression levels of jnk‐1 and sir‐2.1 were significantly elevated (Figure 4X–Z). In conclusion, vitamin K2 may modulate the stress response in *C. elegans* and extend its lifespan via the jnk‐1/sir‐2.1/daf‐16 signaling cascade.

## 4 Discussion

Vitamin K2 is identified as a fat‐soluble Vitamin present in various food sources including meat, eggs, dairy products like yogurt and milk, as well as fermented foods such as soy (natto) and cheese (Dunlop et al. 2023). The primary mechanisms of action of Vitamin K in disease management are its anti‐inflammatory and anti‐oxidative properties (Xie et al. 2024). Vitamin K exhibits beneficial effects on conditions such as asthma (Saglani et al. 2023), cancer (Bouzahzah et al. 1995), and neurodegenerative diseases (Zhang and Yan 2023; da Silva et al. 2013). Given the superior performance of Vitamin K2 as an antioxidant, we investigated the potential mechanisms through which Vitamin K2 mitigates stress levels and consequently extends lifespan.

In our study, we found that Vitamin K2 can extend the lifespan and enhance the health of *C. elegans* . However, this does not imply that Vitamin K2 is free from potential risks. Our prior research demonstrated that the effects of Vitamin K2 on *C. elegans* are dose‐dependent. Specifically, a concentration of 10 μM Vitamin K2 reduced the lifespan of *C. elegans* , whereas concentrations of 1 μM and 5 μM significantly increased its lifespan. Given that 1 μM Vitamin K2 exhibited inferior performance compared to 5 μM Vitamin K2 in follow‐up experiments, the latter concentration was selected for further investigations.

The aging process is frequently associated with alterations in physiological states, including the gradual decline in muscle cell viability, which results in diminished motility in *C. elegans* (da Silva et al. 2013). The deceleration of the pharyngeal pumping rate with advancing age not only signifies a reduction in the nematode's food intake but may also elicit the DR effect (Ryu et al. 2016). Numerous studies have demonstrated that anti‐aging interventions can significantly enhance these aforementioned parameters (Fang et al. 2017; Lin et al. 2019). Intestinal integrity is intricately linked to the aging process in *C. elegans* , and numerous studies have demonstrated that intestinal permeability progressively increases as *C. elegans* age (Dambroise et al. 2016; McGee et al. 2011). Lipofuscin, an autofluorescent marker indicative of nematode senescence, progressively accumulates with advancing age (Liang et al. 2023). Our findings demonstrate that Vitamin K2 supplementation not only significantly extends the lifespan of *C. elegans* but also mitigates the accumulation of age‐related lipofuscin. These results align with those observed for numerous other natural extracts and bioactive compounds (Liang et al. 2023; Sun et al. 2023, 2021). In conclusion, Vitamin K2 has been demonstrated to extend the lifespan of *C. elegans* and enhance its normal physiological functions.

Some studies attribute the primary role of vitamin K2 in promoting longevity to its regulation of lipid metabolism rather than to its influence on oxidative stress (Qu et al. 2022).

However, the capacity to withstand stress is closely associated with the aging process (Xie et al. 2024). Aging is frequently associated with inflammation and oxidative stress, which exacerbate each other, creating a vicious cycle (Vatner et al. 2020). Oxidative stress is closely linked to mitochondrial function, and mitochondrial dysfunction may serve as a significant initiating factor in the aging process (López‐Otin et al. 2023). Throughout the aging process, mitochondria experience varying degrees of oxidative damage, leading to impaired energy metabolism, cellular dysfunction, and ultimately cell death (Burtscher et al. 2023). This study demonstrates that vitamin K2 exhibits significant stress‐regulatory properties. By preserving mitochondrial morphology and modulating the mitochondrial oxidative respiratory chain, vitamin K2 reduces reactive oxygen species (ROS) levels in aging *C. elegans* , thereby enhancing their resistance to oxidative stress.

The PMK‐1/p38‐mediated stress signal pathway plays a critical role in *C. elegans* , influencing the health, aging and innate immunity of *C. elegans* (Liu, Wang, Zhu, et al. 2022; Soltanmohammadi et al. 2022). The key downstream molecule Skn‐1 is the direct *C. elegans* ortholog of the mammalian Nrf/CNC family of proteins (Blackwell et al. 2015; Glover‐Cutter et al. 2013), and its role in regulating oxidative stress defense mechanisms has been extensively investigated. Moreover, Skn‐1 plays a critical role in mediating the mitochondrial unfolded protein response in *C. elegans* (Glover‐Cutter et al. 2013). Our findings demonstrate that although vitamin K2 enhances the expression of key molecules—sek‐1, nsy‐1, and pmk‐1—within the PMK‐1/p38 signaling pathway in *C. elegans* , its effects on stress resistance and lifespan extension are independent of this pathway. Instead, the transcription factor skn‐1 appears to play a pivotal role in mediating the anti‐stress effects and longevity‐promoting properties of vitamin K2. This indicates that skn‐1 might be a more downstream or independent node, yet the mechanistic association remains unclear. skn—1 is a well—recognized longevity factor. Activation of both daf—16 and skn—1 has been observed in numerous nematode mutants associated with longevity (Liu, Wang, Zhang, et al. 2022; Zhang et al. 2025). Several studies have also demonstrated that skn‐ 1 plays a role in regulating daf—16, and the activation of skn—1 and daf—16 is closely linked to stress regulation and aging (Deng et al. 2020). The specific mechanism through which skn—1 may utilize vitamin K2 to extend lifespan in Caenorhabditis will be the focus of our future research.

ROS have the potential to perturb the normal physiological state of cells. The cellular response to elevated levels of ROS generally encompasses the activation of numerous intracellular signal pathways, including the mitogen‐activated protein kinase (MAPK) family (Torres and Forman 2003). Specifically, c‐Jun N‐terminal kinase 1 (JNK1), a member of the MAPK family, assumes a pivotal role in signal transduction mediated by growth factors, cytokines, and various cellular stresses, such as heat shock and ROS (Torres and Forman 2003; Bode and Dong 2007). Sir‐2.1 is the direct homolog of mammalian Sirt‐1 in *C. elegans* . Sirt‐1, an NAD‐dependent deacetylase, plays a regulatory role in multiple pathways, including those involved in stress protection (Nasrin et al. 2009). Oxidative stress modulates *sirt‐1* activity via *jnk‐1* by influencing its subcellular localization and functional status (Nasrin et al. 2009). Furthermore, *daf‐16* is essential for the *sir‐2.1*‐mediated extension of lifespan (Tissenbaum and Guarente 2001). *Daf‐16* not only extends the lifespan of *C. elegans* but also enhances their stress resistance (Mukhopadhyay et al. 2006). Enhancing the transcriptional activity of *daf‐16* further promotes stress resistance (Lin et al. 2018). Genes targeted by *daf‐16* include the stress response genes *sod‐3* and *hsp‐16.2*, which serve as indicators of *daf‐16*'s transcriptional activity. Elevated expression of *sod‐3* and *hsp‐16.2* can mitigate oxidative stress in *C. elegans* (Muñoz 2003; Murphy 2006). Additionally, skn‐1 can be transcriptionally activated by daf‐16 (Onken and Driscoll 2010). The increased expression of skn‐1 observed in our study may be mediated through the jnk/sir‐2.1/daf‐16 pathway.

Collectively, Vitamin K2 has been demonstrated to effectively mitigate mitochondrial stress in *C. elegans* , thereby extending its lifespan and enhancing its physiological condition.

It is important to note that our study was performed exclusively in *C. elegans* , and although this model is valuable for dissecting fundamental aging mechanisms, it does not fully recapitulate complex age‐related pathologies such as Alzheimer's disease or cardiovascular dysfunction. Therefore, while our results suggest that vitamin K_2_ promotes healthy aging in *C. elegans* , extrapolation to human diseases should be made with caution. Future studies using transgenic nematode strains expressing human disease‐associated proteins (e.g., Aβ, α‐synuclein) or vertebrate models will be essential to determine whether vitamin K_2_ exerts protective effects in specific disease contexts.

To clarify, our study was carried out solely on one species, *C. elegans* . Although this species is useful for aging research, it fails to fully replicate the complexity of mammalian physiology and age—related pathologies. Consequently, any generalizations of our findings to humans should be made with caution. In the future, validation using mammalian cell lines and vertebrate models like mice will assist in determining whether the anti—aging effects of vitamin K_2_ observed in this study are generalizable and in evaluating its potential for treating age—related diseases.

## 5 Conclusions

Collectively, our results demonstrate that Vitamin K2 extends lifespan and improves mitochondrial function in *C. elegans* through the JNK‐1/SIR‐2.1/DAF‐16 pathway. While these findings highlight the potential of Vitamin K2 as an anti‐aging intervention, further studies using vertebrate models and specific age‐related disease models are necessary to evaluate its translational relevance.

## Author Contributions

Song‐Yu Guo and Shao Li: Conceptualization; Song‐Yu Guo: Data curation; Song‐Yu Guo and Shao Li: Formal analysis; Shao Li: Funding acquisition; Song‐Yu Guo, Yu‐Qi Li, Wen‐Fei Zheng, Hua‐Piao, Si‐Qi Li, Ze‐Yang Liu, Jun‐Ting Lv, Yue Kong, Qi‐Fa Li, Ying‐Zi Wang, Shu‐Zhuang Li, Chun‐Li Zhao and Shao Li: Methodology; Shao Li: Project administration; Shu‐Zhuang Li: Resources; Song‐Yu Guo and Yu‐Qi Li: Validation; Song‐Yu Guo: Visualization; Song‐Yu Guo and Shao Li: Writing – original draft; Song‐Yu Guo and Shao Li: Writing – review and editing.

## Funding

This work was supported and funded by the National Natural Sciences Foundation of China (82471464,82401823,82101661) and the Leading Talent Team Project of the Education Department of Liaoning Province (LJ222510161002) and DMU&DLBL 2026JCYJ‐02.

## Ethics Statement

The authors have nothing to report.

## Consent

The authors have nothing to report.

## Conflicts of Interest

The authors declare no conflicts of interest.

## Supporting information


**Figure S1:** (A) The accumulation levels of reactive oxygen species (ROS) in nematodes after a 10—day supplementation with 1 μM, 5 μM, and 10 μM vitamin K2. (B) Statistical analysis of reactive oxygen species (ROS) fluorescence intensity in nematodes following the administration of 1 μM, 5 μM, and 10 μM vitamin K2 for 10 days (C) The adenosine triphosphate (ATP) content in *Caenorhabditis elegans* after a 10—day supplementation with 1 μM, 5 μM, and 10 μM vitamin K2 (D) Mitochondrial morphology in *C. elegans* following the administration of 1 μM, 5 μM, and 10 μM vitamin K2 for 10 day (E) Statistics on mitochondrial roundness following a 10—day administration of 1 μM, 5 μM, and 10 μM vitamin K2 to *C. elegans*. One‐way ANOVA was used for comparisons among multiple groups. Values are presented as mean ± SEM; All of these measurements were made at least three times. *p < 0.05, **p < 0.01, ***p < 0.001.
**Figure S2:** (A) Motility statistics of N2, VC199, VC8, and CF1038 *C. elegans* on the first day of adulthood. (B) Statistics on the pumping rates of pharyngeal pumps in N2, VC199, VC8, and CF1038 *C. elegans* on the first day of adulthood (C) The oviposition count of N2, VC199, VC8, and CF1038 *C. elegans* (D) Statistical analysis of the body lengths of N2, VC199, VC8, and CF1038 strains of *C. elegans*. One‐way ANOVA was used for comparisons among multiple groups. Values are presented as mean ± SEM; All of these measurements were made at least three times. *p < 0.05, **p < 0.01, ***p < 0.001.

## Data Availability

The data that support the findings of this study are available on request from the corresponding author. The data are not publicly available due to privacy or ethical restrictions.
