# Supplementary material for: Vitamin K2 Extends Lifespan by Alleviating Mitochondrial Stress via the JNK‐1/SIR‐2.1/DAF‐16 Signaling Axis in Caenorhabditis elegans
Source: Aging Cell. 2026 May 3;25(5):e70530. doi: 10.1111/acel.70530 (PMC13135748; doi:10.1111/acel.70530)
Supplement: Supplementary file 1 — Figure S1: (A) The accumulation levels of reactive oxygen species (ROS) in nematodes after a 10—day supplementation with 1 μM, 5 μM, and 10 μM vitamin K2. (B) Statistical analysis of reactive oxygen species (ROS) fluorescence intensity in nematodes following the administration of 1 μM, 5 μM, and 10 μM vitamin K2 for 10 days (C) The adenosine triphosphate (ATP) content in Caenorhabditis elegans after a 10—day supplementation with 1 μM, 5 μM, and 10 μM vitamin K2 (D) Mitochondrial morphology in C. elegans following the administration of 1 μM, 5 μM, and 10 μM vitamin K2 for 10 day (E) Statistics on mitochondrial roundness following a 10—day administration of 1 μM, 5 μM, and 10 μM vitamin K2 to C. elegans. One‐way ANOVA was used for comparisons among multiple groups. Values are presented as mean ± SEM; All of these measurements were made at least three times. *p < 0.05, **p < 0.01, ***p < 0.001. Figure S2: (A) Motility statistics of N2, VC199, VC8, and CF1038 C. elegans on the first day of adulthood. (B) Statistics on the pumping rates of pharyngeal pumps in N2, VC199, VC8, and CF1038 C. elegans on the first day of adulthood (C) The oviposition count of N2, VC199, VC8, and CF1038 C. elegans (D) Statistical analysis of the body lengths of N2, VC199, VC8, and CF1038 strains of C. elegans. One‐way ANOVA was used for comparisons among multiple groups. Values are presented as mean ± SEM; All of these measurements were made at least three times. *p < 0.05, **p < 0.01, ***p < 0.001. [file ACEL-25-e70530-s001.docx]

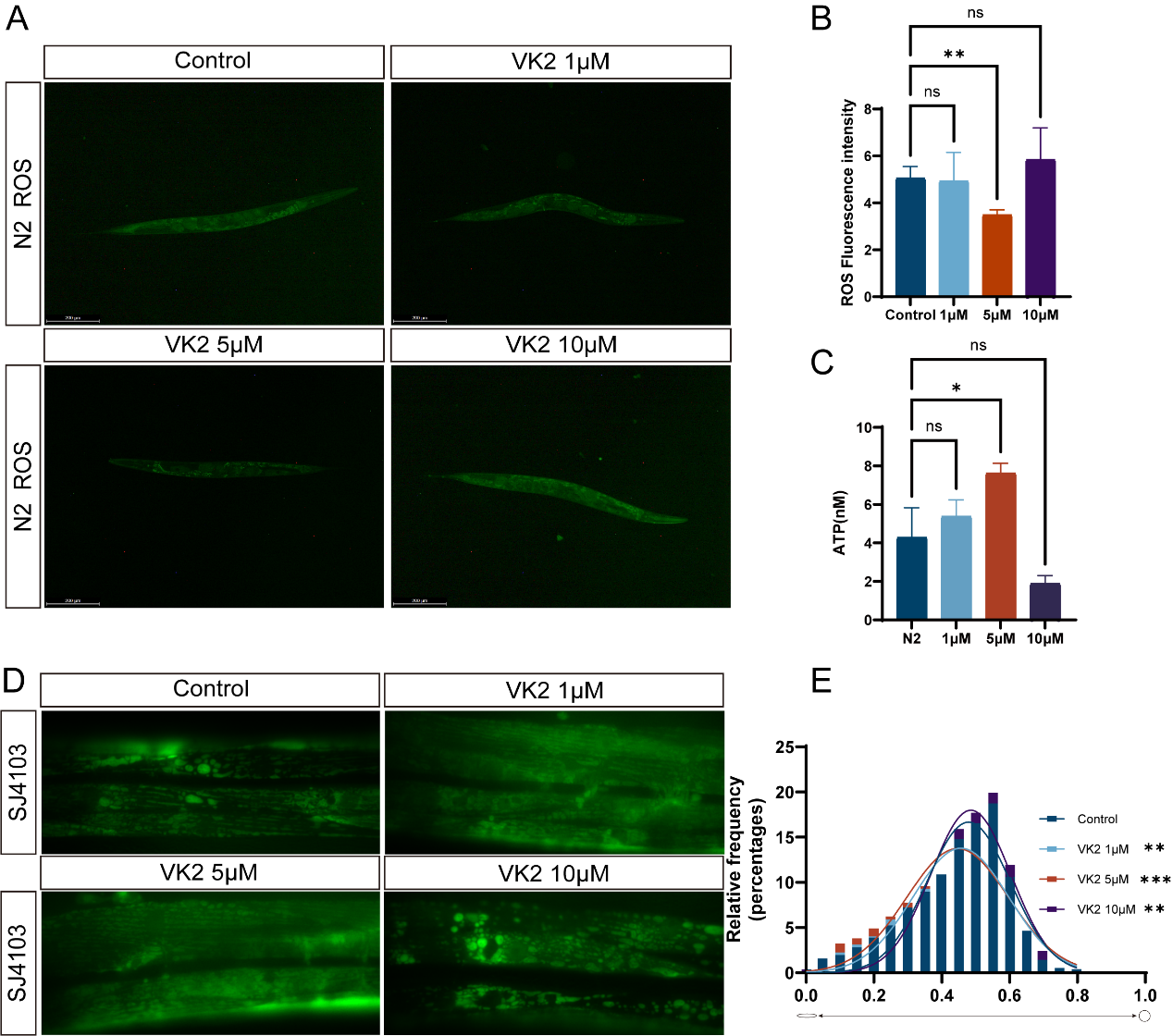


**Fig. S1.** (A) The accumulation levels of reactive oxygen species (ROS) in nematodes after a 10 - day supplementation with 1μM, 5μM, and 10μM vitamin K2. (B) Statistical analysis of reactive oxygen species (ROS) fluorescence intensity in nematodes following the administration of 1 μM, 5 μM, and 10 μM vitamin K2 for 10 days (C) The adenosine triphosphate (ATP) content in Caenorhabditis elegans after a 10 - day supplementation with 1μM, 5μM, and 10μM vitamin K2 (D) Mitochondrial morphology in *C.elegans* following the administration of 1μM, 5μM, and 10μM vitamin K2 for 10 day (E) Statistics on mitochondrial roundness following a 10 - day administration of 1 μM, 5 μM, and 10 μM vitamin K2 to *C.elegans*. One-way ANOVA was used for comparisons among multiple groups. Values are presented as mean ± SEM; All of these measurements were made at least three times. **p* < 0.05, ***p* < 0.01, ****p* < 0.001.


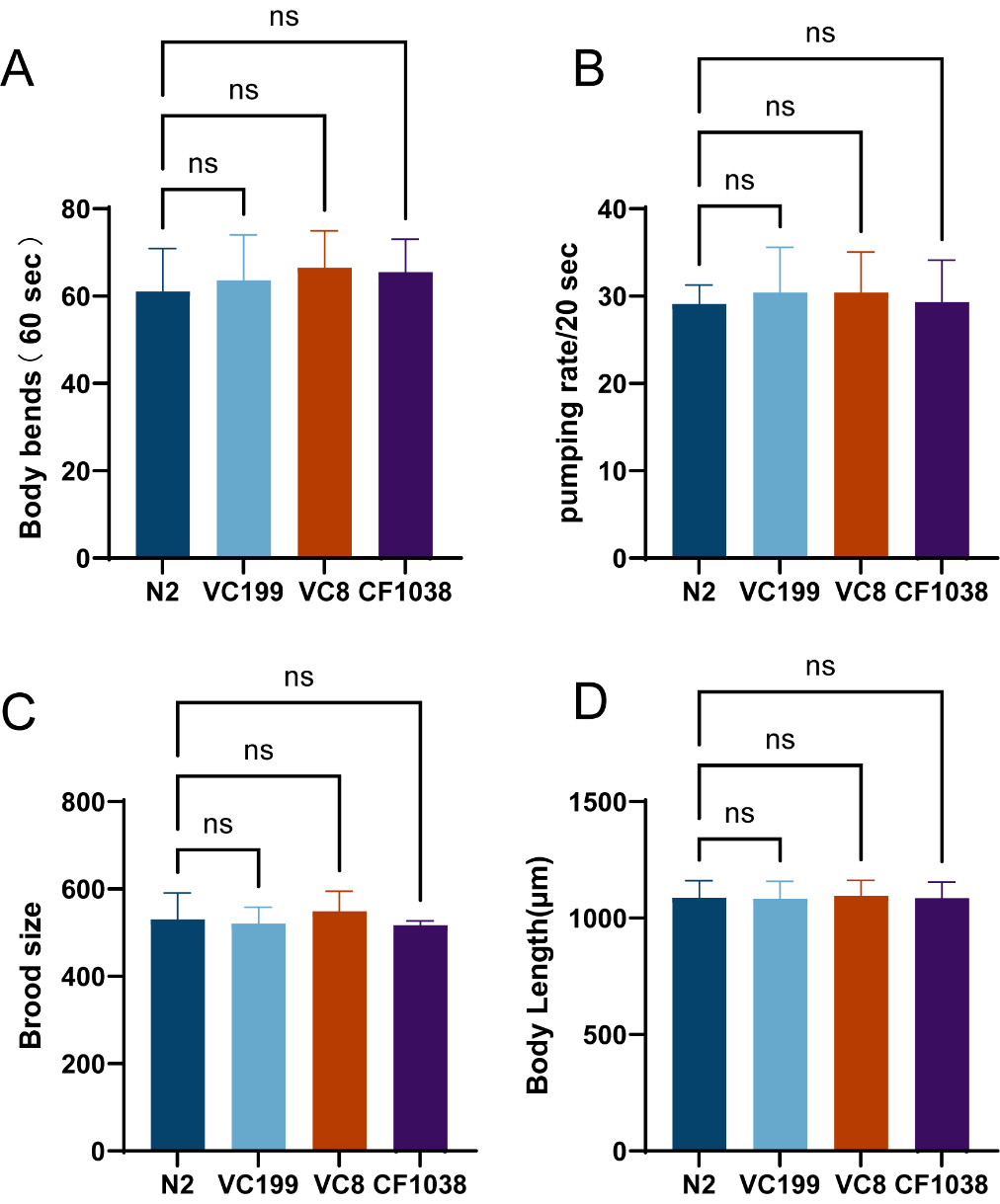


**Fig. S2.** (A) Motility statistics of N2, VC199, VC8, and CF1038 *C.elegans* on the first day of adulthood. (B) Statistics on the pumping rates of pharyngeal pumps in N2, VC199, VC8, and CF1038 *C.elegans* on the first day of adulthood (C) The oviposition count of N2, VC199, VC8, and CF1038 *C.elegans* (D) Statistical analysis of the body lengths of N2, VC199, VC8, and CF1038 strains of *C.elegans.* One-way ANOVA was used for comparisons among multiple groups. Values are presented as mean ± SEM; All of these measurements were made at least three times. **p* < 0.05, ***p* < 0.01, ****p* < 0.001.
